# Efficient Image Processing Technique for Detecting Spatio-Temporal Erosion in Boron Nitride Exposed to Iodine Plasma

**DOI:** 10.3390/nano15130961

**Published:** 2025-06-21

**Authors:** Ahmed S. Afifi, Janith Weerasinghe, Karthika Prasad, Igor Levchenko, Katia Alexander

**Affiliations:** 1 School of Engineering, College of Systems and Society, The Australian National University, Canberra, ACT 2601, Australia; ahmed.afifi@anu.edu.au (A.S.A.); karthika.prasad@anu.edu.au (K.P.); katia.bazaka@anu.edu.au (K.A.); 2 Plasma Sources and Application Centre, NIE, Nanyang Technological University, Singapore 637616, Singapore; levchenko.igor@nie.edu.sg

**Keywords:** plasma–material interaction, boron nitride erosion, iodine and argon plasma, image registration algorithm, erosion mapping, surface morphology evolution

## Abstract

Erosion detection in materials exposed to plasma-generated species, such as those used for space propulsion systems, is critical for ensuring their reliability and longevity. This study introduces an efficient image processing technique to monitor the evolution of the erosion depth in boron nitride (BN) subjected to multiple cycles of iodine plasma exposure. Utilising atomic force microscopy (AFM) images from both untreated and treated BN samples, the technique uses a modified semi-automated image registration method that accurately aligns surface profiles—even after substantial erosion—and overcomes challenges related to changes in the eroded surface features. The registered images are then processed through frequency-domain subtraction to visualise and quantify erosion depth. Our technique tracks changes across the BN surface at multiple spatial locations and generates erosion maps at exposure durations of 24, 48, 72 and 84 min using both one-stage and multi-stage registration methods. These maps not only reveal localised material loss (up to 5.5 μm after 84 min) and assess its uniformity but also indicate potential re-deposition of etched material and redistribution across the surface through mechanisms such as diffusion. By analysing areas with higher elevations and observing plasma-treated samples over time, we notice that these elevated regions—initially the most affected—gradually decrease in size and height, while overall erosion depth increases. Progressive surface smoothing is observed with increasing iodine plasma exposure, as quantified by AFM-based erosion mapping. Notably, up to 89.3% of surface heights were concentrated near the mean after 72–84 min of plasma treatment, indicating a more even distribution of surface features compared to the untreated surface. Iodine plasma was compared to argon plasma to distinguish material loss during degradation between these two mechanisms. Iodine plasma causes more aggressive and spatially selective erosion, strongly influenced by initial surface morphology, whereas argon plasma results in milder and more uniform surface changes. Additional scale-dependent slope and curvature analyses confirm that iodine rapidly smooths fine features, whereas argon better preserves surface sharpness over time. Tracking such sharpness is critical for maintaining the fine structures essential to the fabrication of modern semiconductor components. Overall, this image processing tool offers a powerful and adaptable method for accurately assessing surface degradation and morphological changes in materials used in plasma-facing and space propulsion environments.

## 1. Introduction

Small satellites and CubeSats are increasingly becoming the technology of choice for a wide range of missions, from communication to space exploration, with their utility closely linked to the development of miniaturised systems that can deliver the required level of performance and operational lifetime within very limited volume, mass and power budgets [1]. One research area that has received a significant amount of attention is the development of new miniaturised electric propulsion systems, as they promise to provide sufficient thrust and specific impulses to enable satellite orbit changes and other manoeuvres while operating within the required power limitations. Electric propulsion systems capable of operating effectively using propellants other than xenon are of particular interest, given the expensive and relatively scarce nature of the latter, and the requirement for a pressurised storage tank that contributes to the system mass and volume, increases the complexity of integration with other satellite systems and raises safety concerns. While noble gases such as argon or krypton are less expensive and more abundant, their performance is inferior to that of xenon and their use still requires the presence of a storage tank. In contrast, propellants such as iodine or the aromatic hydrocarbons fluorobenzene and naphthalene [2,3] can be stored in their solid form and undergo solid/liquid-vapor phase transition during low-power sublimation, eliminating the need for high-pressure systems [4,5,6,7]. As a much cheaper alternative, they also offer lower ionisation energies compared to xenon, and, in the case of aromatic hydrocarbons, lower post-ionisation fragmentation when compared to other presently studied molecular propellants. However, hydrocarbons are generally considered incompatible with materials used in, e.g., high-current hollow cathode thermionic emitters, devices used to supply electrons for plasma ionisation and to neutralise the exhaust, as they decompose into high chemically reactive atomic components at the temperatures at which the thermionic material is kept during emission, potentially leading to surface modification and compromising the performance of the material. Furthermore, they offer lower density when compared to both high-pressure xenon and such solid propellants as iodine, with the latter offering a density three times that of xenon [8].

Due to its high chemical reactivity, the use of iodine as a propellant presents a set of unique challenges for both the development of the propulsion system and its subsequent integration into the satellite [3,4]. These challenges become even more pronounced in thrusters operating at power levels above 100 kW [2]. Despite these difficulties, companies like Busek are actively advancing iodine-based Hall thruster technology [9]. Unlike xenon, the impact of exposure to iodine species, both ionised and un-ionised, on the performance and lifetime of components, e.g., solar panels, spacecraft structures and sensitive instruments, has not been fully explored, and the effects of chemical erosion and iodine adsorption/absorption on a variety of surfaces require thorough investigation. While erosion-induced changes to surface roughness can damage optical coatings or radiator emissivity, the accumulation of iodine species on, e.g., dielectric surfaces can lead to shorts. Furthermore, erosion of the plasma-interfacing elements of the propulsion system itself, particularly in electric propulsion systems like ion thrusters and Hall effect thrusters, can lead to a reduction in their longevity and efficiency.

As such, accurate erosion detection and modelling are crucial for ensuring material compatibility and understanding potential mechanisms of failure. This, in turn, relies on our ability to collect high-quality data pertaining to material loss and changes in the surface geometry, which can be more challenging for in situ measurements. Typical techniques used to measure erosion on surfaces exposed to ion bombardment include profilometry [10,11], telemicroscopy [12,13], laser-induced fluorescence (LIF) [14,15] and quartz crystal microbalance (QCM) [16]. Optical and laser-based erosion measurement techniques such as those described in [12] are also used to measure both radial and axial erosion parameters in, e.g., gridded ion and Hall effect thrusters. A system known as Cavity Ring-Down Spectroscopy (CRDS) has been designed to monitor the erosion of boron nitride (BN), a typical material used to line the acceleration channel of the Hall effect thruster, in real-time [17]. The methods used to measure the erosion rate in a Hall thruster using spectroscopy include laser absorption spectroscopy [18], laser-induced breakdown spectroscopy [19], continuous-wave cavity ring-down spectroscopy [17,20] and optical emission spectroscopy (OES) [21]. Table 1 presents a tabular comparison of different erosion monitoring techniques, highlighting the challenges of these methods, including the complexity of the setup and the limited understanding of the erosion process dynamics.

When studying the progression of erosion depth, it is essential to explore how inherent material non-uniformities affect subsequent plasma–surface interactions [22,23]. This understanding is key to understanding the link between, e.g., thruster efficiency and surface geometry and to developing the concept of self-healing propulsion systems through tracking the evolution of erosion depth [24]. In particular, capturing and analysing the spatio-temporal evolution of surface morphology under plasma exposure is essential for understanding how surface features evolve and influence plasma–surface interactions over time. Non-uniform surface charging can cause plasma-generated particles—such as ions—to concentrate more on specific morphological features of the surface, depending on its shape or structure. This preferential targeting enhances erosion non-uniformity, which not only affects the lifetime and reliability of components in Hall thrusters but may also alter the dominant failure mechanisms. A clear understanding of these processes is essential not only for accurate predictive modelling but also for guiding the development of strategies for healing and plasma-enabled surface repair.

This article aims to model the spatio-temporal evolution of surface morphology to better understand the erosion dynamics, material volume loss and the mechanisms of failure. It investigates the use of image processing techniques, specifically image registration and subtraction, as an effective strategy for monitoring the evolution of surface morphology as it is exposed to plasma-generated species. In this work, we specifically focus on the corrosive effect of iodine plasma on boron nitride, a material used for the walls of Hall thrusters; however, we also successfully apply the same approach to study the effect of argon plasma, with Ar used as a propellant, on boron nitride, as we will show later.

**Table 1:** Comparison of erosion monitoring techniques.

Technique	Accuracy	Spatial Resolution	Ease of Use	Notes
Cavity Ring-Down Spectroscopy (CRDS) [25]	High sensitivity to minute erosion species	No spatial resolution (volume-averaged concentration within the cavity)	Complex setup (requires active cavity alignment due to thermal drift)	Ideal for quantifying ablation rates; unsuitable for analysing fine surface morphology.
Optical Emission Spectroscopy (OES)	Moderate accuracy [25]	Line-of-sight only; spatial mapping requires multi-fibre setup [26]	Simple setup but requires precise plasma state knowledge for quantification	Real-time monitoring based on excited species emission; unsuitable for analysing fine surface morphology.
Quartz Crystal Microbalance (QCM)	High sensitivity to mass changes	No spatial resolution (measures a single point representing the average mass change on the crystal surface)	Simple setup; measures only deposition without identifying specific materials	Provides only surface-averaged data rather than localised measurements; unsuitable for analysing fine surface morphology.
Laser Profilometry [27,28]	Moderate to high (limited by laser line width and surface reflectivity)	Limited by laser spot size	Moderate setup (requires calibration and adjustment for different surface dimensions)	Contactless 3D surface reconstruction; affected by shadows, vibrations and thermal drift; suitable for analysing fine surface morphology
AFM-based Image Processing (Our technique)	High (nanometre-scale)	High (sub-micron to nanometre)	Simple setup (AFM only); post-processing required (can be automated)	Enables detailed visualisation and quantification of surface erosion evolution; well-suited for analysing fine surface morphology.

Here, image registration is used to align multiple images of the same scene into a common coordinate system and is an important step before performing image subtraction. This alignment is essential for accurate comparison and analysis, especially for atomic force microscopy (AFM). It allows us to monitor the evolution of depth and shape profile of the same erosion areas over time of exposure. Importantly, it allows for the integration of multiple visualisation and characterisation modalities, which may be helpful in understanding the spatio-temporal differences in the erosion processes. For example, in this study [29], two imaging modalities, namely AFM and optical microscopy, were integrated using a cross-scale image registration method based on geometrical patterns by identifying and matching geometrical features common to both types of images. In other studies [30,31], an improved alignment between scanning electron microscopy (SEM) and AFM images was achieved using different approaches. Geldmann et al. [30] used Monte Carlo simulations to generate multiple SEM images based on the AFM height profile and then used the simulated images to refine the registration through an optimisation process, while Wortmann et al. [31] used two local feature extraction algorithms, namely a scale-invariant feature transform (SIFT) and speeded-up robust features (SURF), for the registration of AFM and SEM images. As shown by Wang et al. and Fan et al., AFM images collected at different resolutions [32] and in different operational modes [33] can be successfully used for registration.

Once registered, image subtraction can be used to highlight the differences between the images collected at different time points of plasma exposure, providing a useful means for the qualitative and quantitative estimation of erosion over different areas of the surface. Image subtraction is a widely used technique in various image processing fields, including astronomical observations [34,35], medical imaging [36] and remote sensing research [37].

In our study, it is essential to confirm if image registration remains feasible despite significant erosion, as this may reduce the likelihood of finding matching geometric features on heavily eroded images, particularly since erosion by argon is milder compared to iodine. Here, we present an efficient erosion imaging monitoring technique. In Section 2, the materials and methods used are presented. Section 3 discusses the steps of the image processing technique and the image registration methods applied to images before and after treatment. Section 4 and Section 5 cover the discussion and conclusion.

## 2. Materials and Methods

The level of iodine exposure varies depending on the location within the spacecraft. Storage tanks are subjected to the highest partial pressure of iodine, while external surfaces experience the lowest. The inner walls, which interact directly with the iodine plasma plume, are exposed to a partial pressure of 0.1 Pa during thruster operation [8]. This partial pressure was selected because iodine particles are most energetic and aggressive in terms of corrosion, making plasma–wall interactions the primary focus of this study.

It is important to note that in the storage tank, iodine is not ionised; it is most likely stored as a solid and then sublimated, which is a key advantage of using it as a propellant. In the thruster channel, iodine becomes ionised and accelerated, increasing the damage from collisions with the walls. Once outside the thruster, the iodine, which has been neutralised, is exposed to radiation and interactions with atomic oxygen.

Electric propulsion systems can operate for months or even years (Table 2). The National Institute of Standards and Technology (NIST) database [38] was used to obtain iodine’s partial pressure. For the simulation, we tested four durations lasting from six months to three and half years of thruster operation. To conduct accelerated testing, we used an approximation proposed by Zschatzsch et al. [39] which considers that no chemical reaction occurs between the chemically inert gas mixture of argon (Ar) and iodine ($I_{2}$). As a result, both gases can be described individually by the ideal gas equation:

(1) $$pV=nk_{B}T$$

where p is the partial pressure of the gas, V is the volume of reservoir containing the gas mixture, n is the number of particles, k_B_ is the Boltzmann constant and T is the absolute temperature. This assumption ensures that the gas mixture behaves predictably and adheres to fundamental thermodynamic principles.

The number of particle interactions per unit area, or the collision density $J_{collision}$, can be calculated using the following formula, assuming the system behaves as an ideal gas [39].

(2) $$J_{collision}=\frac{p}{4k_{B}T}.\sqrt{\frac{8k_{B}T}{\pi M}}$$

where p the partial pressure of iodine and M is the molecular mass of iodine. Since the partial pressure of iodine varies with temperature, the required temperature values to achieve the desired partial pressure values, were obtained from the NIST database [40]. To maintain a constant temperature, the iodine reservoir was heated to the target temperature using a positive temperature coefficient (PTC) heating element.

The methodology investigated the reactivity of iodine under controlled conditions, focusing on partial pressure, temperature and exposure duration. A constant temperature was maintained throughout the experiment to eliminate variations in reaction kinetics. The ideal gas assumption, as described in Equation (1), simplified calculations and modelling by ensuring no interactions occur between Ar and $I_{2}$ [39]. This study followed an exposure equivalence model, assuming that the exposure effect (E) is proportional to the product of partial pressure (p) and exposure duration (Δt), expressed as E∝p⋅Δt. This principle enabled an accelerated simulation of long-term exposure by adjusting pressure and time parameters accordingly. As a result, maintaining a constant p⋅Δt ensured that varying pressure and exposure duration under simulated conditions yielded the same effect as in space.

**Figure 1:**
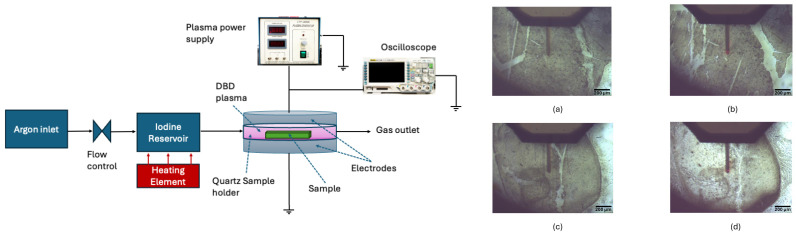
Schematic diagram of the DBD plasma setup used for the accelerated testing. Optical images of BN samples during the alignment stage of AFM imaging. (**a**,**b**) Images of Sample 1 before ((**a**), initial image) and after (**b**) 84 min of plasma treatment. (**c**,**d**) Images of Sample 2 before ((**c**), initial image) and after (**d**) 72 min of plasma treatment.

The laboratory setup, as illustrated in Figure 1, utilised ultra-high purity argon gas (99.997%, BOC) as the carrier gas for the experiment. The flow rate of the argon was set at 5 L/min to ensure uniform dielectric barrier discharge (DBD) plasma throughout the chamber. This gas was passed through an iodine (Sigma Aldrich, St. Louis, MO, USA) reservoir maintained at 80 °C. The resulting iodine and argon gas mixture was then directed into a quartz DBD chamber, where two samples, each with an area of $1\;{\mathrm{cm}}^{2}$, were placed in the centre of the plasma zone. Argon was utilised to isolate the samples and iodine from air and humidity, as iodine is hygroscopic, and to facilitate plasma generation. BN films were prepared on 1 mm thick soda–lime glass substrates (Sail) as follows. The substrates were first ultrasonically cleaned in 95% ethanol (Wilmar Sugar, Proserpine, Australia) for 5 min and then dried under ambient conditions. Next, the glass substrates were immersed in BN suspension (Sigma Aldrich) and then spin coated using a KW-4A spin coater (Setcas LLC, San Diego, CA, USA) at 3000 rpm for 130 s, followed by 4000 rpm for 30 s. The coated substrates were then placed on a hot plate heated to 150 °C for 15 min and allowed to cool to ambient conditions.

Figure 1a,c show the AFM cantilever positioned for scanning initial (pretreated) BN Samples 1 and 2, respectively, placed in the quartz DBD chamber alternately. Samples 1 and 2 were exposed to iodine plasma under identical conditions, with maximum exposure times of 84 and 72 min as shown in (b) and (d), respectively. However, visible initial surface variations between them highlight inherent material inhomogeneity. This non-uniformity prior to plasma exposure helps explain the localised and uneven erosion observed post-treatment.

Our approach employed a multilayer coating method that combines dip-coating and spin-coating, as outlined above. This method was inspired by the work of Cho et al. [41], who developed multilayer coatings by alternating BN with marker metals like silver. These marker layers are placed to track erosion progression through their unique spectroscopic emissions during HT operation. Their research showed that embedding these multilayer coatings into the channel walls of an HT allows for precise erosion rate measurements. This is achieved by monitoring the emission signals from the marker layers as they are exposed during the sputtering process caused by plasma interactions. This study utilised ion beam sputtering to deposit the multilayer coatings, presenting a new approach to evaluating material performance and durability in high-energy plasma environments.

The CTP 2000 plasma system (ACS Material LLC, Pasadena, CA, USA) was used to generate the necessary voltage to create plasma, and the electrical characteristics were recorded using an MSO1104 oscilloscope (Rigol, Portland, OR, USA). The plasma parameters for the simulation experiment were as follows: peak-to-peak voltage of 25 kV, plasma current of 0.1 mA, frequency of 9.1 kHz, argon as the carrier gas and a flow rate of 5 L/min.

## 3. Image Processing Technique

**Figure 2:**
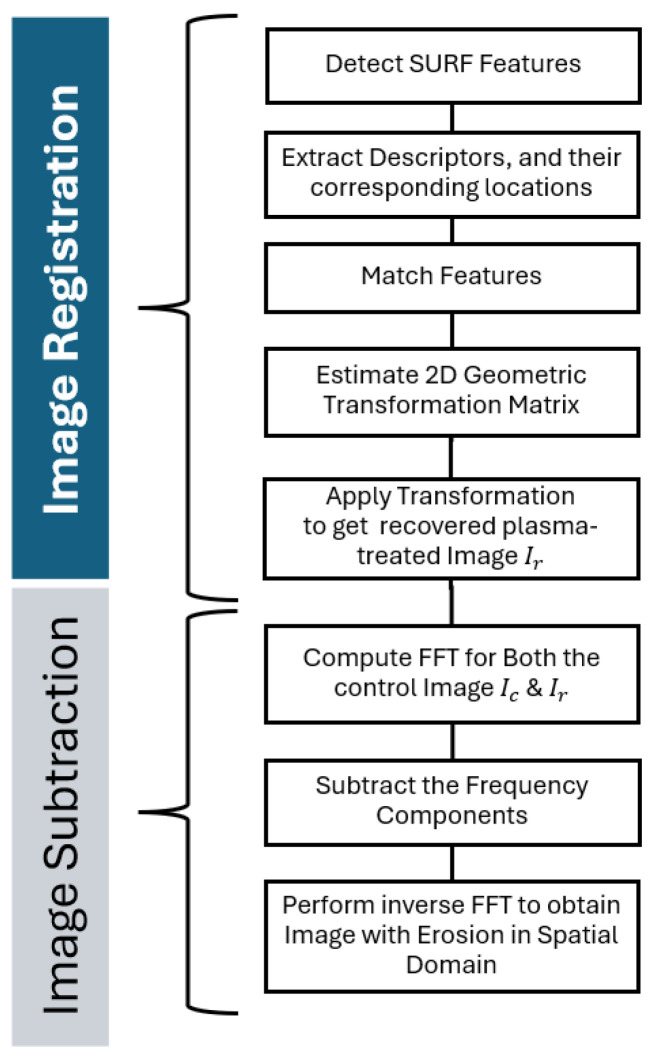
Flowchart of image processing.

The flowchart details a two-part image processing technique consisting of image registration and image subtraction, as shown in Figure 2. The image registration part includes SURF detection, feature extraction, feature matching and estimating the transformation matrix to find the recovered plasma-treated image ($I_{r}$). The image subtraction part involves computing the fast Fourier transform (FFT) for both the initial image ($I_{c}$) and ($I_{r}$), followed by subtracting frequency ($I_{c}-I_{r}$) to detect the difference in the resultant image then performing an inverse FFT to obtain the erosion depth image in the spatial domain.

### 3.1. Image Registration

The technique used for image registration is a modified semi-automated feature matching method. The main technique identifies and matches key features between different images using the SURF detection algorithm [42], which is well-suited for real-time applications. Feature descriptors [43] characterise the detected features, and then are compared to find correspondences between features in different images. The nearest neighbour algorithm is used to improve the accuracy of feature matches [44].

This technique is particularly effective for aligning AFM images due to its low computational cost in detecting and describing thousands of interest points. These points are simultaneously extracted from each pair of AFM images—captured before and after the sample’s exposure to plasma at varying durations. With longer exposure times, the extent of erosion is expected to be more significant, deforming the image of plasma-treated samples to the point where it may become difficult if not impossible to reliably use the SURF automation technique. As such, we chose to use a semi-automated method that manually matches at least two features from each AFM image, even if it was not extracted automatically. The aim of the proposed method is to find the pairwise correspondence between AFM images of initial and plasma-treated samples using their spatial relations or various descriptors of features. We chose SURF for detecting features in AFM images because it consistently delivers reliable results, outperforming other common methods like SIFT [45], BRISK [46], Harris [47] and FAST [48]. SURF’s strength comes from its use of Haar wavelet responses, which handle the complex textures of AFM data better than SIFT. We also found that intensity-based image registration usually aligns AFM images more accurately than SIFT, but SURF has the added benefit of allowing semi-automated adjustments when matching points are scarce—something intensity-based methods cannot easily achieve, especially when surface erosion is severe.

There are challenges in image registration techniques for rigidly transformed images with significant distortion and deformation. First, distortions caused by high erosion of BN, as evident in the image for the plasma-treated sample, are a major source of misregistration. This misregistration can occur due to image differences that come from unmodeled and intrinsic variations [49]. Unmodeled variations may result from factors during image acquisition such as changes in lighting conditions, significant erosion affecting differences in pixel intensities and sensor-specific artifacts (e.g., AFM introducing unique artifacts like strip noise). On the other hand, intrinsic variations represent genuine differences between images due to changes in the underlying content. Examples include scene changes and feature occlusions caused by erosion in plasma-treated AFM images, as well as viewpoint variations when capturing AFM images at the micrometre level. For instance, when images are captured from different viewpoints of a scene with surfaces that have significant depth variations, the distortions will include occlusions that vary between images, objects appearing in different relative positions and other more localised distortions.

### 3.2. Image Subtraction in Frequency Domain

Image subtraction is a fundamental technique in image processing used for detecting changes between two images. A subtraction image is created by subtracting the grey values of corresponding pixels from two images after they have been aligned through image registration. The grey value in the subtraction image indicates the degree of change between the two images.

To apply image subtraction in the frequency domain, we first use the fast Fourier transform (FFT) to convert both images from the spatial domain to the frequency domain. Then, we perform subtraction in the frequency domain and finally convert the result back to the spatial domain using an inverse FFT. The output image, which represents the difference between the initial and the recovered plasma-treated images, is the erosion depth image.

Using the Fourier space (or frequency domain) for image subtraction offers several advantages over the spatial domain, namely filtering the noise including the periodic noise which appears as repetitive patterns [50] and improved computational performance.

### 3.3. Image Filtering

The noise reduction is important before image registration to enhance features and improve accuracy and consistency in AFM images. To estimate erosion depth from the AFM images of samples before and after plasma exposure, we set the proportional and integral gains of the scanning process to fixed values. Noise introduced during scanning can interfere with the registration accuracy and appear as erosion in the output erosion depth image.

Filtering helps to remove this noise, making the images more suitable for precise alignment and more easily used for identifying and matching key points between images during registration. This improves registration accuracy and determines output erosion depths. The removal of AFM artifacts, such as strip noise and tip-related distortions, can be performed in either spatial or frequency domain [51]. Preserving edge sharpness, particularly along internal regions where pixel intensity changes sharply, rather than at the image boundaries, can also be achieved by denoising in the frequency domain.

There are multiple methods used to perform noise reduction. One technique which we use in our study is using the NanoSurf CoreAFM softwarev3.10.5 filtering technique, which provides advanced algorithms to reduce noise while preserving important features. It uses a 3 × 3 Gaussian convolution filter which can be applied to the AFM measurements. Whether smoothing or sharpening is applied during image filtering, it is essential to preserve the original data as much as possible.

The Laplacian Gaussian filter method focuses on both edge detection and noise reduction. The method first uses a Gaussian filter for smoothing the noisy image and then uses a Laplacian filter for edge detection within the image, specifically those internal regions where pixel intensity shifts occur sharply, which are important for feature extraction. The denoising is necessary because the Laplacian filter has a tendency for amplifying the noise when dealing with noisy images. This filtering technique is applied on both the initial AFM images and the AFM images of samples after the plasma treatment prior to the image registration process.

AFM introduces striping noise that impacts the output image, manifesting as high-amplitude erosion depths. Removing this noise is important in detecting the actual range of erosion at each specific plasma exposure time. The Gwyddion softwarev2.66 [52] can be used effectively to denoise AFM scanned images, removing most of the scars and stripes introduced during the scanning process.

### 3.4. Direct and Indirect Methods

**Figure 3:**
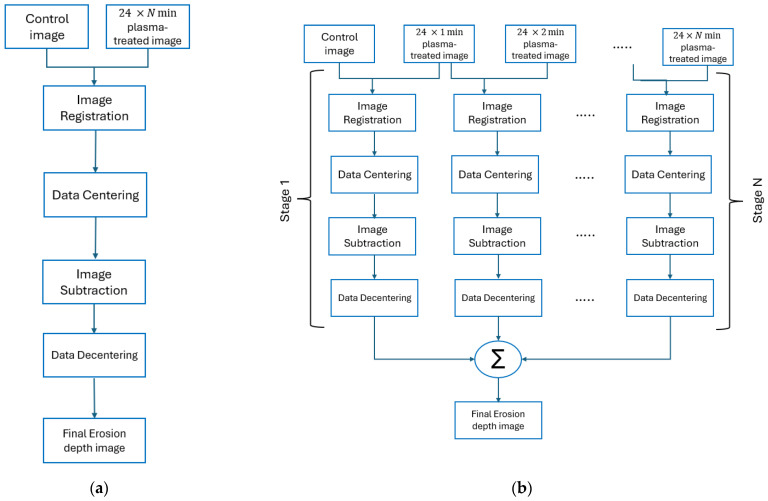
Two techniques used to obtain the final erosion depth image: (**a**) one-stage method and (**b**) multi-stage method. The initial (control) image refers to the image collected for the sample prior to plasma treatment. The plasma-treated image refers to the image(s) collected for the same sample at different stages of plasma treatment at the intervals of 24 min. Arrows indicate the sequential processing flow from the control and plasma-treated image(s) to the final erosion depth image.

Figure 3 illustrates two methods for obtaining the final erosion depth image: the direct (one-stage) method and the indirect (multi-stage) method. Direct Approach (One-Stage Method): This method starts with the image registration techniques being used to align the initial image, with the images collected after the sample was exposed to plasma for 24 × N min, where N can be 1, 2, 3, or 3.5. After the alignment, the algorithm centres the data by calculating and centring the centre of each AFM image to a common reference point. Next, image subtraction is performed in the frequency domain. Both the initial and the centred registered images are transformed using the fast Fourier transform (FFT), and their frequency components are subtracted to highlight differences between the images of the sample before and after plasma treatment. The inverse FFT is then applied to obtain the erosion depth image in the spatial domain. Finally, data decentralisation is performed, reversing the centralisation process to achieve the final erosion depth image. For instance, to determine the erosion after 84 min of treatment using the one-stage approach, we compare the image at 84 min with the initial image at 0 min, which provides the final erosion depth.

Indirect Approach (Multi-Stage Method): This method has a potential advantage in determining surface degradation between successive plasma-treated surfaces and is expected to deliver similar overall erosion estimates to the direct method. It involves multiple stages of the direct approach, as illustrated in Figure 3. We begin by applying the direct approach to successive pairs of images collected at 24 min intervals starting from 0 min and then at 24 min, 48 min, 72 min and 84 min. Erosion depth is computed between each pair. For instance, the image at 24 min is compared with the initial image at 0 min estimate the erosion for the 0–24 min period. Next, the image at 48 min is compared with the one at 24 min (now the initial for this stage) to determine erosion for the 24–48 min period. This process is then repeated for the remaining intervals within the 48–84 min period. Summing these erosion values should yield height variations similar to those obtained by directly comparing the image at 84 min with the initial image at 0 min. This method enables more accurate identification of the same geometrical surface features, as they are likely to degrade less in 24 min than they are in 84 min, where much of the original topography might be lost. The final 24*N plasma-treated image is considered as N stages of a 24 min plasma-treated image, distributed as depicted in Figure 3. In each stage, the output of data decentring is accumulated to obtain the final erosion depth image.

In AFM data analysis, we select the raw *z*-axis data from the forward scan, which consists of a 2D matrix with z-components representing the heights of the scanned sample at specific locations. The outputs of the initial images and those images collected subsequent to plasma treatment have different centres in the chart data range. The centre $C_{C,P}$ of either the initial image ($I_{c}$) or the image of the plasma-treated sample ($I_{P}$) is calculated using the following formula:

(3) $$C_{C,P}=\max\left(I_{c,P}\right)+\left(\frac{-S_{C,P}}{2}\right)$$

where $S_{C,P}=\left|\min\left(I_{c,P}\right)-\max\left(I_{c,P}\right)\right|$ is the span of either the image of the initial sample or the plasma-treated sample. Here, $I_{c}$ is the output matrix of the AFM scanned initial image and $I_{P}$ is the output matrix of the AFM scanned image of the sample subsequent to plasma treatment.

We use Equation (3) to compute the centres of the line graph of all image pixels of both ($I_{c}$) and ($I_{P}$). Then, the pixels of ($I_{P}$) are shifted from the centre ($C_{P}$) to the centre ($C_{C}$) of the initial image to establish a common reference point before performing image subtraction.

## 4. Results and Discussion

### 4.1. Surface Evolution Under Iodine Plasma

**Figure 4:**
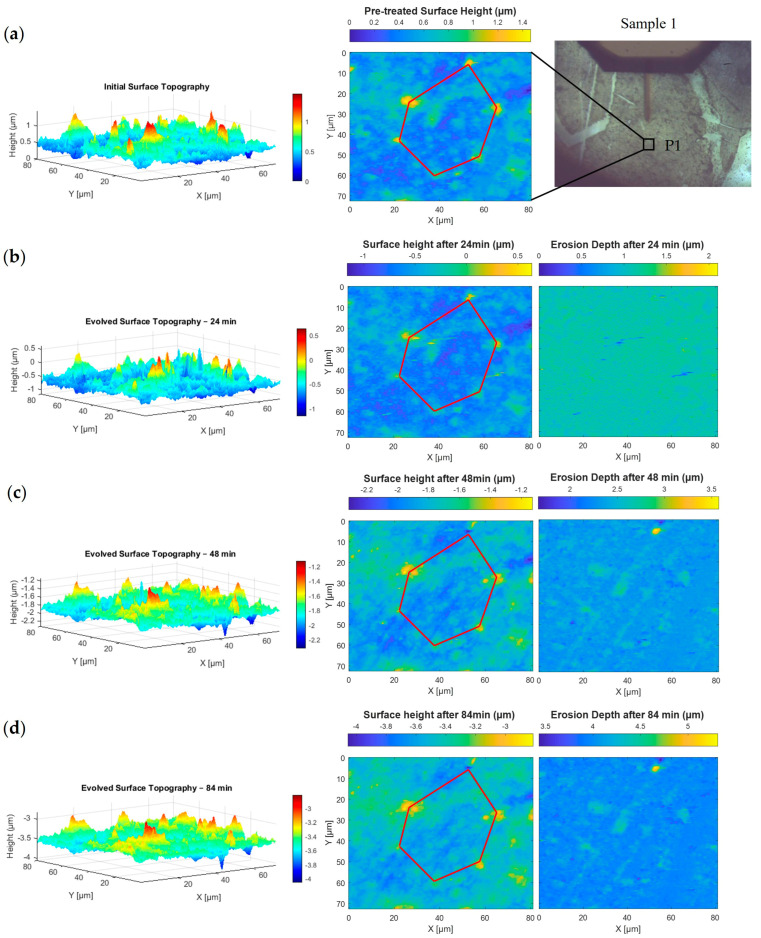
Surface evolution and erosion mapping of BN Sample 1 at position P1 under iodine plasma exposure for 0, 24, 48 and 84 min. (**a**) Initial (pretreated) 3D and 2D surface topography with the exact analysed region highlighted. (**b**) After 24 min: Aligned AFM data shows early topographic changes and erosion depth from spectral height differences. (**c**) At 48 min: Continued surface flattening and reduction in peak features. (**d**) At 84 min: Further surface smoothing, with gradual fading of elevated regions at red polygon vertices.

**Figure 5:**
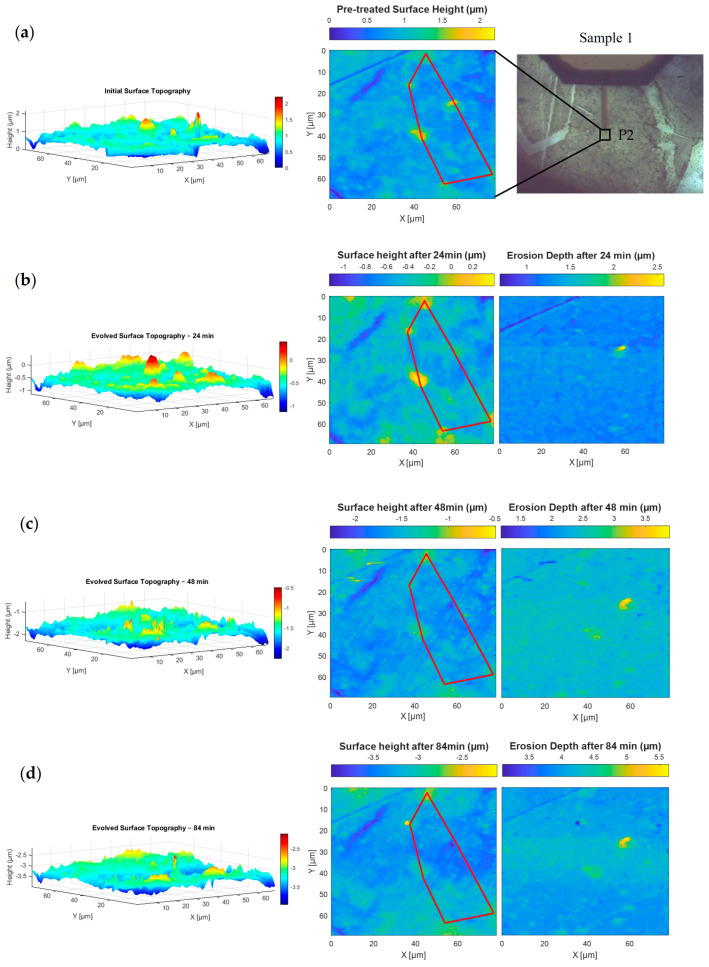
Surface topography evolution and erosion mapping of BN Sample 1 at position P2 under iodine plasma exposure for 0, 24, 48 and 84 min. (**a**) The pretreated 3D and 2D maps show fewer surface peaks compared to position P1. (**b**) After 24 min, aligned AFM data reveal changes in topography and an apparent increase in peak features due to an overall reduction in surface height. (**c**) At 48 min, the surface undergoes noticeable smoothing and flattening, clearly visible in both 3D and 2D views. (**d**) By 84 min, further smoothing is observed, with prominent elevated regions gradually fading at red polygon vertices.

**Figure 6:**
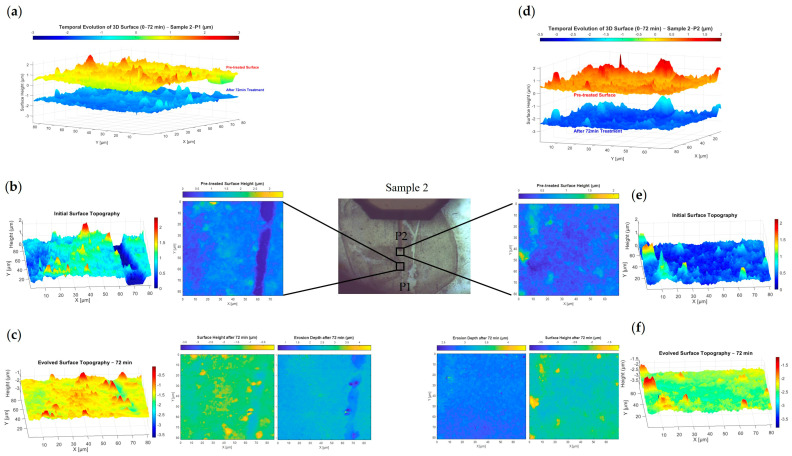
Surface topography evolution and erosion mapping of BN Sample 2 at positions P1 and P2 under iodine plasma exposure, comparing pre-treatment (0 min) and post-treatment (72 min) states using a one-stage method. (**a**) Three-dimensional overlay of the surface at P1 before and after 72 min of plasma treatment, highlighting topographic changes. (**b**) Initial 3D and 2D topography maps at P1 (t = 0 min) and (**c**) the corresponding evolved surface and erosion depth maps after 72 min, showing the effects of plasma exposure. (**d**) Three-dimensional overlay of pretreated and treated surfaces at P2, showing changes after 72 min. (**e**) Initial 3D and 2D maps at P2 (t = 0 min) and (**f**) the corresponding evolved maps at 72 min, used to visualise roughness evolution and compute erosion depth.

This study, as summarised in Table 2, investigates four exposure durations—D1 through D4—to simulate space environmental conditions involving varying levels of iodine plasma interaction with the material. In D1, the erosion analysis is performed using the direct (one-stage) method, with results shown in Figure 4b and Figure 5b for positions P1 and P2, respectively. Each position is scanned via AFM at a resolution of 90 μm × 90 μm, illustrating how erosion features develop in different surface areas of the same sample. D2 employs both the direct and indirect (multi-stage) methods to map erosion depth, although image alignment becomes more complex due to the multiple registration steps involved. The corresponding topographic and erosion maps for P1 and P2 are shown in Figure 4c and Figure 5c. In D3, Sample 2 undergoes 72 min of plasma exposure, with the direct method used to produce erosion depth images, as seen in Figure 6. Finally, D4 applies both mapping methods, incorporating previous erosion rates from D1 (24 min) and D2 (48 min) to refine the erosion depth analysis, as shown in Figure 4d and Figure 5d.

Figure 4 shows how the BN surface at position P1 on Sample 1 evolves over time under iodine plasma exposure at intervals of 0, 24, 48 and 84 min. The left column shows 3D topography highlighting surface roughness, the middle column provides corresponding 2D height maps and the right column displays erosion depth calculated by comparing each state to the initial surface. A red polygon marks a fixed region used to consistently track surface changes. Its vertices, typically shown in yellow, indicate areas of high elevation that are more susceptible to erosion.

Initially, in Figure 4a, the surface displays sharp peaks, particularly at the vertices of the red polygon. After 24 min (Figure 4b), there is an apparent increase in the number of visible peaks in the 3D and 2D views. This is likely due to an overall reduction in surface height, which makes smaller topographic features stand out more clearly. Another possible factor is the presence of residual AFM artifacts or surface scars, some of which remain noticeable in the 2D height map. However, the majority of these artifacts were minimised or removed during the preprocessing steps described earlier. By 48 min (Figure 4c), the peaks start to flatten noticeably. After 84 min (Figure 4d), significant smoothing is observed as elevated features diminish further in both height and number. Together, these visualisations highlight the progressive and localised nature of BN surface erosion under iodine plasma treatment.

Figure 5 presents the evolution of the BN surface at position P2 on Sample 1 under iodine plasma exposure, observed at four different time intervals: 0, 24, 48 and 84 min. Each row corresponds to a specific time point, displaying the 3D surface topography (left), 2D height map (middle) and erosion depth map (right). The red polygon outlines a fixed region used to track changes consistently over time. A visual of the sample with the marked position P2 is also provided for reference.

In the initial state (Figure 5a), the pretreated surface at P2 shows fewer and less pronounced peaks compared to P1, indicating a relatively smoother starting texture. After 24 min (Figure 5b), early signs of erosion appear with a slight reduction in surface height. At 48 min (Figure 5c), the surface undergoes noticeable smoothing, with the peak heights reducing further and the surface becoming more uniform. By 84 min (Figure 5d), a further reduction in surface roughness is observed, with most elevated regions—especially those at the red polygon vertices—visibly faded or flattened. For instance, one of the vertices at the top of the red shape decreases from approximately 1.3 µm to an average of −2.8 µm, resulting in an erosion rate of 4.1 µm after 84 min, as shown in Figure 5d. These results reflect the progressive erosion and surface homogenisation due to extended plasma exposure.

Figure 6 illustrates the surface topography evolution of BN Sample 2 at two distinct positions (P1 and P2) before and after 72 min of iodine plasma exposure using a one-stage treatment approach. At position P1, panel (a) presents a side-by-side 3D overlay of the surface before and after treatment, showing a clear reduction in surface height and roughness. Panels (b) and (c) provide detailed 3D and 2D visualisations of the surface at t = 0 and after 72 min, respectively, along with erosion depth maps that quantify material removal and highlight significant surface smoothing.

Similarly, at position P2, panel (d) compares pre- and post-treatment surfaces in 3D, revealing a marked decrease in height and topographic features. Panels (e) and (f) display the initial and evolved surface maps at this location, again showing reduced peak prominence and more uniform surface characteristics after plasma exposure.

**Figure 7:**
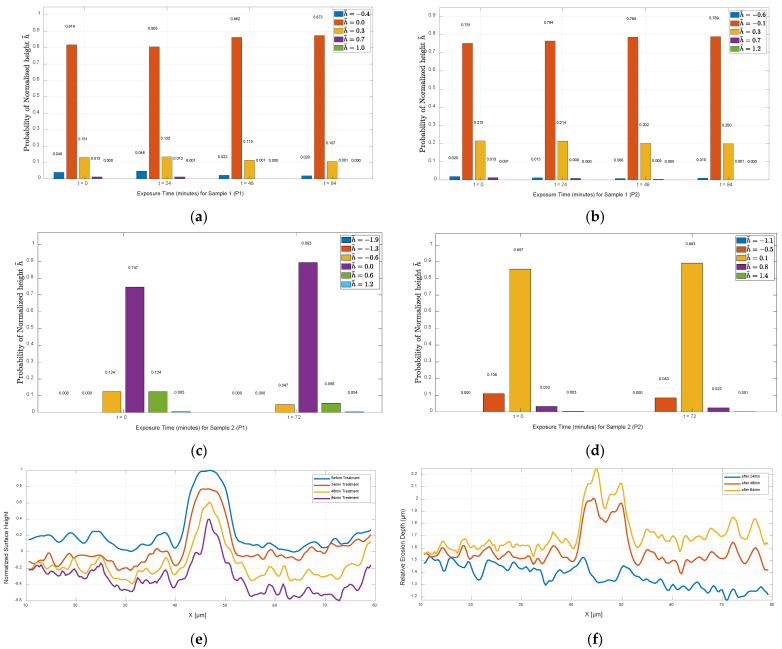
Normalised surface height distributions (mean-adjusted to h = 0) illustrating the evolution of surface smoothness with increasing plasma exposure time for (**a**) Sample 1 at position P1, (**b**) Sample 1 at position P2, (**c**) Sample 2 at position P1 and (**d**) Sample 2 at position P2. A higher percentage around zero indicates a trend toward surface homogenisation. Panels (**e**,**f**) show the spatio-temporal evolution of surface morphology under iodine plasma exposure: (**e**) normalised surface height profiles for Sample 1 and (**f**) relative erosion depth after 24, 48 and 84 min.

Figure 7 presents bar charts illustrating the evolution of surface height distributions over time under plasma exposure for Sample 1 and Sample 2 at positions P1 and P2. To enable comparison, surface height data at various time points (0, 24, 48, 72 and 84 min) were first normalised by subtracting their mean values, thereby centring each dataset around zero. Specifically, each AFM height matrix $h_{t}$ at time t was normalised as

(4) $$h_{t}^{norm}(i,j)=h_{t}(i,j)-\frac{1}{MN}\sum_{i=1}^{M}\sum_{j=1}^{N}h_{t}(i,j)$$

where $h_{t}\left(i,j\right)$ is the height element at row i and column j of the matrix $h_{t}$ and M and N are the number of rows and columns of the height matrix, respectively.

This mean-centring process ensured that all datasets were aligned around zero without altering their relative spread, allowing for consistent binning and comparison across time. A common bin range was then calculated using the global minimum and maximum values across all normalised datasets within each sample. These edges defined uniform bins used for all histograms, with the number of bins *n* (typically 5 or 6) chosen based on which provided better visual representation. The frequency counts within each bin were normalised to probabilities using

(5) $$P_{t}\left(h_{b}\right)=\frac{N_{t}(h_{b})}{\sum_{k=1}^{n}N_{t}(h_{k})}$$

where $h_{b}$ is the center of the b-th bin, $N_{t}(h_{b})$ is the count of normalised height values $h_{t}^{norm}(i,j)$ falling within the bin centered at $h_{b}$ and n is the total number of bins.

Each resulting probability distribution was then visualised as a grouped bar chart with exposure time on the *x*-axis. Each group represents a specific time point, and each bar colour corresponds to a bin centred on a particular normalised height. Numerical labels on the bars display the exact associated probabilities. These charts help evaluate how surface roughness progresses toward uniformity, as indicated by increasing concentrations of height values near zero over time.

As observed in Figure 4, the surface of Sample 1 at position P1 undergoes progressive smoothing following iodine plasma exposure, particularly after 48 and 84 min. This trend is quantified in Figure 7a, which shows that 81.6% of the untreated surface heights fall within the range of −0.216 μm to 0.142 μm (represented by the orange bar). After 24 min of exposure, this percentage slightly decreases to 80.5%, indicating a temporary drop in uniformity as explained previously. However, as exposure continues, the percentage rises to 86.2% at 48 min and reaches 87.3% at 84 min, confirming a steady increase in surface uniformity. Correspondingly, the probabilities in the outer bins (−0.575 μm to −0.216 μm and 0.142 μm to 0.501 μm), which represent surface peaks and valleys, decrease over time. This reflects the gradual flattening of surface features, consistent with the 3D topographic changes shown in Figure 4.

Looking at the second chart in Figure 7b, which displays the probabilities of normalised surface heights for Sample 1 at position P2, a clear trend of increasing surface uniformity is observed. The percentage of surface heights concentrated around the mean value—within the range of −0.343 μm to 0.092 μm, centred at −0.12 μm (represented by the orange bar)—rises progressively from 75.1% in the untreated state to 76.4%, 78.5% and 78.9% after 24, 48 and 84 min of plasma exposure, respectively. This is accompanied by a steady decline in the proportion of surface heights in the adjacent bins, which represent peaks and valleys, from 21.5% to 21.4%, then to 20.2%, and finally 20%, indicating a gradual reduction in surface irregularities over time. As Sample 2 was subject to iodine plasma exposure for 72 min only, Figure 7c,d quantify the surface height distribution for two different positions P1 and P2 (shown in Figure 6), respectively. The key bin centres ($\tilde{h}$) for these positions—which represent the range of surface heights near the mean—are centred at 0 and 0.1 μm, highlighted by the purple and yellow bars, respectively. At position P1, the probability of surface heights falling within this central bin increases from 74.7% to 89.3%, while at position P2, it rises from 85.7% to 89.3%. This indicates a noticeable smoothing of the surface at both positions. These results align with the 3D topography maps in Figure 6, which reveal a reduction in pronounced peaks and valleys after plasma treatment, further supported by the declining probabilities in the adjacent bins.

Figure 7e,f illustrate the spatio-temporal evolution of surface morphology on a boron nitride sample under iodine plasma exposure. Figure 7e shows normalised surface height profiles across a fixed section (X-direction), recorded before treatment and after 24, 48 and 84 min of plasma interaction. Over time, the surface becomes progressively smoother, and the initial nanoscale features diminish, indicating progressive material removal and a reduction in feature size and height. Figure 7f presents the relative erosion depth, capturing the localised material loss at each position along the surface after each treatment time. These depth profiles reveal non-uniform erosion, with certain regions undergoing more extensive loss. Notably, areas with initially higher surface elevations tend to experience greater erosion, highlighting the influence of initial nanoscale features on the spatial distribution of plasma-induced material loss.

Figure 7e,f show the influence of local surface geometry on plasma–surface interactions. As exposure time increases, the initially uniform plasma flux becomes redirected by nanoscale surface variations, such as curvature, resulting in a non-uniform influx of plasma species. This leads to selective erosion where certain morphological features, such as peaks or ridges, receive enhanced ion bombardment and erode more significantly. Tracking the relative erosion depth provides clear evidence of how surface geometry governs erosion dynamics. These insights are important for testing and improving erosion prediction models, and they also support the development of advanced plasma-based repair methods that either make use of or reduce the effects caused by surface shape.

### 4.2. Surface Interaction with Iodine and Argon Plasma: A Comparative View

**Figure 8:**
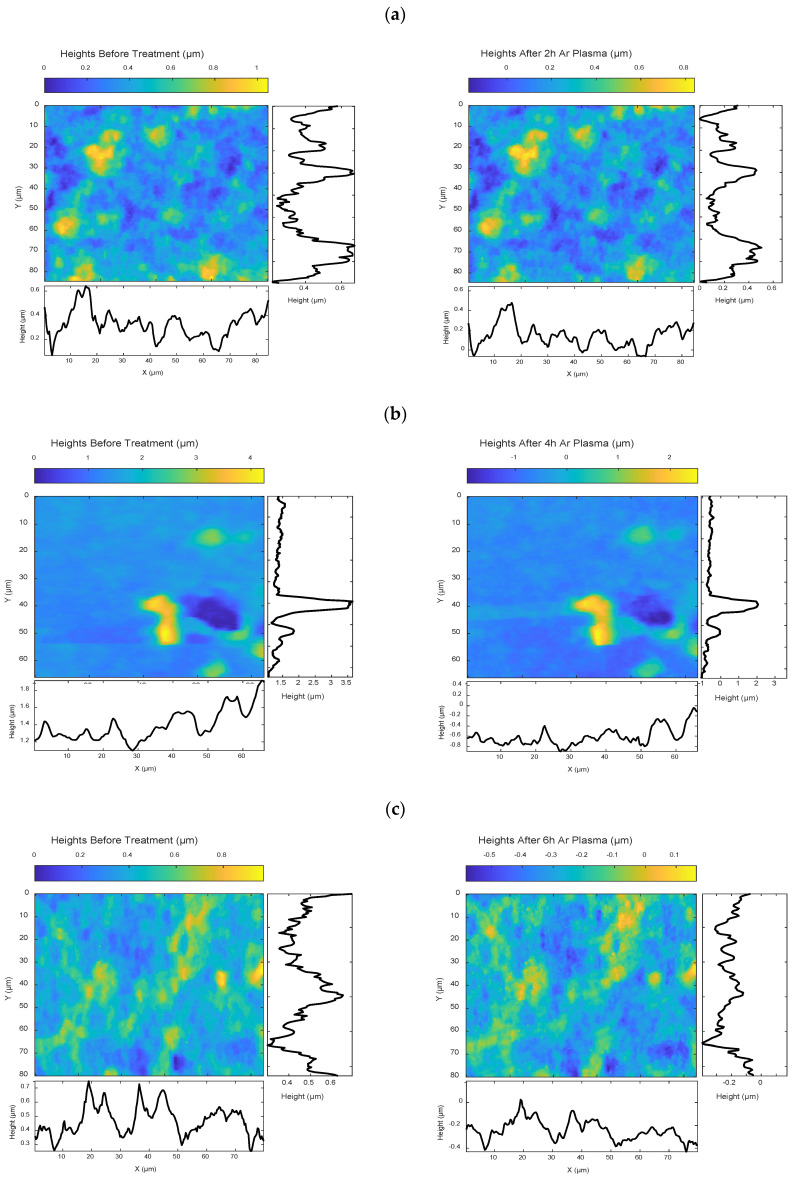
AFM images of three BN samples—untreated and treated with argon plasma for 2, 4 and 6 h—were used to train three artificial neural networks (ANNs) using a Bayesian regularisation training algorithm. The input for the ANNs consists of 1D surface height profiles along both the X and Y directions of the pre-treated surface, while the outputs are the corresponding 1D surface heights after (**a**) 2 h, (**b**) 4 h and (**c**) 6 h of argon plasma exposure.

In this section, we demonstrate how the proposed image processing method can be extended to different erosion systems. As previously shown, iodine plasma—through a combination of ion bombardment and chemical reactions—alters the BN surface over various exposure times (0.4 h, 0.8 h and 1.4 h). We now apply the same image processing approach (described in Section 3) to BN surfaces treated with argon plasma, which involves only physical ion bombardment, to analyse erosion after 2 h, 4 h and 6 h, as illustrated in Figure 8. The objective is to visualise and compare the distinct erosion behaviours driven by these two mechanisms. Argon and xenon are among the most commonly used propellants in space propulsion systems, while iodine is gaining increasing attention due to its high storage density and ease of handling. Recent studies and commercial efforts, such as those by ThrustMe [53], have successfully demonstrated iodine-based propulsion systems in space. Therefore, comparing these plasmas helps to evaluate the material responses under different plasma species, contributing to the design of more resilient propulsion materials tailored to specific candidate propellants.

To make a meaningful comparison, it is essential to start with identical initial surface structures. This is made possible using our proposed method, supported by a machine learning tool that models the behaviours of argon plasma across different BN surfaces and exposure durations. By training this model—specifically an artificial neural network (ANN) using the Bayesian regularisation algorithm—we can predict how the same pre-treated surface (used in the iodine plasma experiments) would respond to argon plasma at equivalent durations. This approach enables a direct, visual comparison between the effects of iodine and argon plasma treatments at their respective exposure times: 0.4 h, 0.8 h and 1.4 h versus 2 h, 4 h and 6 h.

#### 4.2.1. ANN-Based Surface Prediction

To capture the fine spatial variations and evolution of the argon plasma data, we designed three separate ANNs, each corresponding to the end of a specific treatment time. For each network, we extracted 1D surface height profiles along both the X and Y directions, as shown in Figure 8. The input for each network is the 1D height profile of the argon-treated surface before exposure, while the output is the corresponding 1D height profile after treatment.

To prepare the data, we divided the profiles into segments of about 7 μm, containing 20 height measurements each, which set the number of input and output neurons at 20. Each network has one hidden layer with 40 neurons—a size chosen through empirical tuning to balance model complexity with the amount of training data available. The training sets for each network vary due to the registration method explained earlier. To help the model capture the relationships between neighbouring segments, we used a step size equal to half the window length. On average, this produced around 11,000 training samples per network for the 2, 4 and 6 h treatment conditions. In our implementation, the data was automatically divided into 70% for training, 15% for validation and 15% for testing. The maximum number of training epochs was set to 100. The learning rate was not manually specified, as the Bayesian regularisation algorithm used during training dynamically adjusts this parameter throughout the process [54]. Network weights and biases were updated using an optimisation method based on the Levenberg–Marquardt algorithm [55]. The hidden layer uses a hyperbolic tangent sigmoid activation function to capture complex relationships in the data, making it particularly effective for modelling the non-linear behaviour observed in plasma-induced erosion. The output layer uses a linear activation function, which is appropriate for regression tasks involving continuous outputs, such as predicting the temporal surface evolution exposed to argon plasma.

We trained the networks using the Bayesian regularisation algorithm, which minimises a weighted sum of squared errors and weights, improving generalisation by adjusting parameters through backpropagation and adaptive updates until performance improves [56]. The root mean squared errors (RMSE) between the predicted and actual values were 0.066, 0.109 and 0.039, with corresponding R^2^ values of 0.919, 0.941 and 0.891 for NN_2h, NN_4h and NN_6h, respectively, indicating good predictive performance.

#### 4.2.2. Localised Surface Response

**Figure 9:**
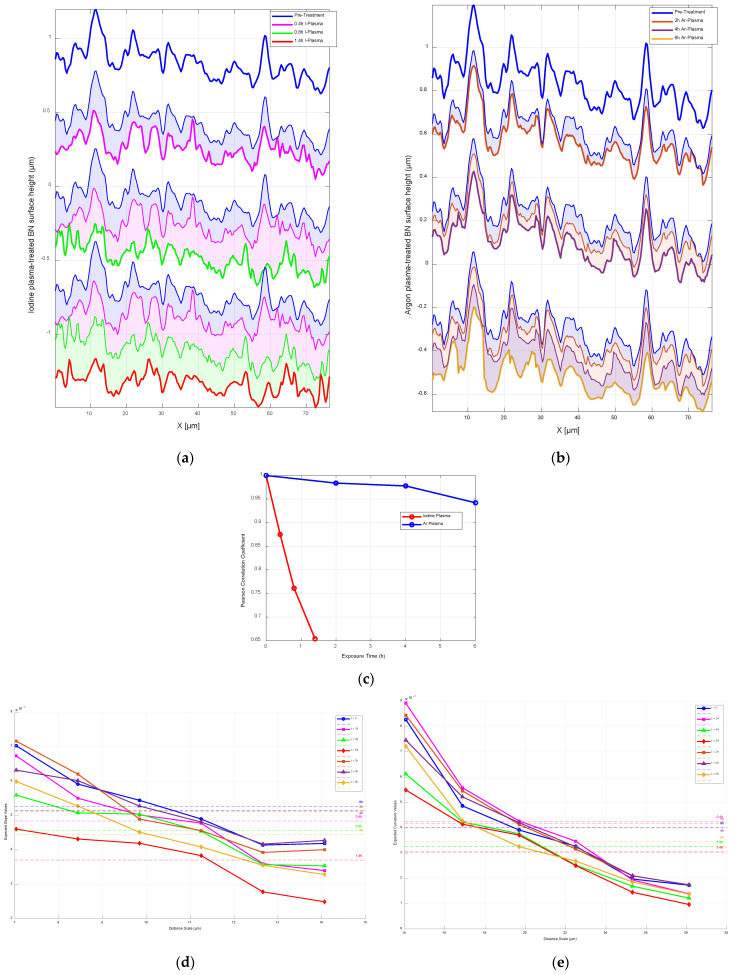
Graphical comparison of BN surface erosion under iodine and argon plasma treatments at discrete exposure times. (**a**) Surface height variations following iodine plasma exposure for 0.4, 0.8 and 1.4 h. (**b**) Predicted surface profiles for argon plasma at 2, 4 and 6 h, generated using three Bayesian neural networks trained on the same pre-treated surface used in (**a**). Shaded regions in different colours indicate the minimum eroded areas between each pair of successive surfaces, with each region adopting the colour of the above surface. (**c**) Correlation coefficients among all six surfaces from (**a**) and (**b**), including the common reference pre-treated surface, with a maximum value of 1. (**d**) Expected slope values of BN surface profiles as a function of distance scale under iodine and argon plasma treatments. (**e**) Expected curvature values of BN surface profiles as a function of distance scale under iodine and argon plasma treatments.

To clearly assess how iodine and argon plasma treatments affect BN surfaces, we focus on two distinct peaks in the pre-treated surface, located at X = 12 μm and X = 59 μm, as shown in Figure 9a,b. Initially, these peaks rise about 0.354 μm and 0.182 μm above the mean surface height, respectively. After 24 min (0.4 h) of iodine plasma exposure (Figure 9a), the peak at 12 μm is reduced by 29% to approximately 0.25 μm, and the peak at 59 μm drops by 24% to around 0.139 μm. At 48 min (0.8 h), the first peak has lost half its original height, while the second falls below the average surface level. By 84 min (1.4 h), the first peak decreases slightly further to about 0.159 μm, and the second remains below the average height, contributing to an overall smoother surface—an effect we will explore further later.

In contrast, as shown in Figure 9b, argon plasma causes much less change—even after 6 h of exposure. The first peak still retains about 90% of its original height, while the second peak drops by around 40%, but the overall shape of the surface is largely preserved. This contrast is highlighted in Figure 9c, where the correlation with the original surface drops to 0.65 after 1.4 h of iodine treatment but stays high at 0.94 after 6 h of argon exposure. Overall, argon plasma has a much milder effect, maintaining the general surface structure, while iodine plasma causes substantial erosion and smoothing of features. Rogers and Branam [57] studied molybdenum erosion under both iodine and argon plasma, finding that iodine plasma caused significantly higher erosion rates, with an approximately exponential increase with temperature, while argon plasma resulted in much lower erosion and better preservation of surface structural integrity.

#### 4.2.3. Scale-Based Surface Roughness Analysis

To better understand the surface characteristics of BN beyond just height loss, we analysed roughness parameters—specifically slopes and curvatures—using the finite difference method. This approach provides valuable insights into how the surface evolves. To incorporate scale into the analysis, we calculated these finite differences at various spatial intervals, as suggested in [58]. We used one-dimensional surface profiles extracted from AFM topographic scans for comparing changes caused by iodine and argon plasma (see Figure 9a,b). This is appropriate since AFM maps are constructed from a series of adjacent line scans.

Let $h_{I}$(x) and $h_{Ar}$(x) represent the surface heights after iodine and argon plasma exposure, respectively, along the x-direction. The first and second discrete derivatives, $h^{\prime }$(x) and $h^{\prime \prime }$(x), represent the slope and curvature of the surface. At each scale interval, we calculated the distributions of these slopes and curvatures.

The scale distances $x_{s}$ were selected with uniform intervals ranging from approximately 7 μm to 14 μm for slope calculations—representing about 1/10 to 1/5 of the total profile length. For curvature, the spacing was doubled to capture broader surface features. For each of these scales, we then computed the expected value E($x_{s}$) of the slope and curvature distributions at scale $x_{s}$ using the formulas

(6) $$E_{slope}(x_{s})=\sum P\left(h^{\prime },x_{s}\right).h^{\prime }(x_{s})$$

(7) $$E_{curvature}(x_{s})=\sum P\left(h^{\prime \prime },x_{s}\right).h^{\prime \prime }(x_{s})$$

where P($h^{\prime },x_{s}$) and P($h^{\prime \prime },x_{s}$) are the probabilities derived from the calculated distributions for the slope and curvature values, respectively, at each scale $x_{s}$.

Figure 9d illustrates the expected slope values (i.e., the first derivative of the surface height profile) calculated at different spatial scales ranging from 7 to 14 μm using the finite difference method. The plot compares the BN surface before treatment (t = 0) with surfaces exposed to iodine plasma for 0.4, 0.8 and 1.4 h and argon plasma for 2, 4 and 6 h. Each curve corresponds to a specific treatment duration, showing how the average slope changes with increasing plasma exposure. The dashed horizontal lines represent the mean slope across all scales for each condition. The figure clearly shows a sharp decrease in slope under iodine plasma, indicating significant surface smoothing and erosion, whereas the argon plasma causes a more moderate and gradual change, preserving much of the original surface structure.

Figure 9e shows how the expected curvature of the BN surface—calculated as the second derivative of the height profile—changes across different spatial scales (from 14 μm to 29 μm) using the finite difference method. The plot compares the curvature values of the untreated surface (t = 0) with those treated using iodine plasma (t = 0.4 h, 0.8 h and 1.4 h) and argon plasma (t = 2 h, 4 h and 6 h). Each line in the graph represents a treatment time and demonstrates how curvature, which reflects the sharpness or roundness of surface features, evolves with scale. As expected, curvature values decrease with increasing scale for all conditions, indicating smoother features over larger distances.

Notably, the iodine-treated surfaces show a more significant drop in curvature values, especially after 1.4 h, suggesting that iodine plasma rapidly smooths out fine surface details. In contrast, surfaces exposed to argon plasma retain higher curvature values over time, particularly at shorter scales, meaning argon treatment causes much less rounding or flattening of the surface. The dashed horizontal lines indicate the average curvature across all scales for each condition. These averages highlight the sharper reduction caused by iodine plasma compared to the more gradual changes caused by argon, reinforcing the conclusion that iodine is more aggressive in altering surface morphology.

## 5. Conclusions

In this study, we developed an efficient image processing technique to monitor erosion in BN material exposed to iodine plasma over time. By using AFM images and combining image registration with image subtraction, we achieved accurate erosion assessment. Our method effectively addressed the challenges posed by significant structural changes due to erosion and demonstrated high sensitivity across various exposure times. Experimental validation under simulated space conditions confirmed the robustness and accuracy of our technique. We tracked changes in the highest elevated areas over time at two positions (P1 and P2) on Sample 1, monitoring six locations per position. All elevated regions gradually faded, their sizes decreased and erosion depth progressively increased, though not at the same rate. For instance, the average erosion depth around the top-centred vertex reached 5 μm for P1 and 4.1 μm for P2 at the maximum exposure time. We calculated an average erosion rate of 2.4 μm per 48 min for BN using the multi-stage method. In comparison, a previous study [59] reported an erosion rate of 1.67 μm per hour for a sample (A0) with a BN concentration of 70 vol%, with discrepancies attributed to differences in material structure and composition, which significantly influence the surface’s response to plasma exposure. The erosion depth analysis across all durations confirms that longer exposure times result in significantly smoother surfaces, with a marked reduction in peaks and valleys. These findings highlight the aggressive effect of iodine plasma treatment on surface features, as supported by both 3D imaging and statistical surface height distributions, demonstrating that iodine plasma is significantly more effective than argon in reshaping surface morphology. Such insights are critical for predictive modelling and for tailoring plasma-based surface treatment or repair strategies, offering a reliable way to optimise the longevity and reliability of materials used with iodine-based plasma propulsion systems.

Future research could extend this technique to other materials and integrate it with real-time monitoring systems for continuous erosion assessment, thereby enhancing the operational efficiency of space propulsion technologies. Firstly, although our current methodology relies on post-exposure AFM imaging, predictive monitoring can be achieved through an erosion model pre-trained on experimental datasets obtained via our proposed image processing technique. This model can be integrated with spacecraft telemetry data (e.g., operating time, current, ion energy) to predict ongoing surface degradation without the need for direct imaging. Future work could also explore the effects of varying plasma parameters, plasma types, or propellant types to further understand erosion behaviour under different operating conditions. Secondly, regarding the implications for material selection, our technique enables high-resolution, spatio-temporally resolved erosion analysis and highlights localised surface behaviours, rather than relying solely on bulk erosion rate measurements used in most conventional methods. This strongly supports the smart selection of plasma-facing materials (generally) and plasma-exposed spacecraft components (specifically), revealing which materials offer more predictable erosion and which are prone to concentrated degradation. Lastly, regarding adaptability, we clarify that the proposed methodology is not limited to BN or a specific plasma type. It is fundamentally based on surface topography analysis using an efficient image processing technique—including AFM imaging, preprocessing, registration and subtraction—which can be applied to any material exhibiting measurable erosion or etching. As long as surface data (e.g., via AFM or other profilometry tools) is available before and after plasma exposure, the technique can be used for materials such as SiC, LaB_6_, graphite and others. Similarly, it is applicable across different plasma environments, including argon, xenon, oxygen, hydrogen, or mixed ion sources, with appropriate calibration. Our study represents a significant advancement in erosion detection for space propulsion materials, setting the stage for future innovations in this field.

## Figures and Tables

**Table 2:** Space and analogue laboratory conditions used for the environmental simulations. Iodine reservoir temperature was maintained at 80 °C for all other scenarios to achieve the required partial pressure.

Durations	Space Conditions		Simulated Conditions	
	Iodine Partial Pressure (Pa)	Δt (Months)	Iodine Partial Pressure (Pa)	Δt (Mins)
D1	0.1	12	2177	24
D2	0.1	24	2177	48
D3	0.1	36	2177	72
D4	0.1	42	2177	84
D1	0.1	12	2177	24

## Data Availability

Data are contained within the article.
